# An assessment of the direct and indirect costs of breast cancer treatment in leading cancer hospitals in Ghana

**DOI:** 10.1371/journal.pone.0301378

**Published:** 2024-05-21

**Authors:** Patience Gyamenah Okyere Asante, Adobea Yaa Owusu, Joseph Ransford Oppong, Kingsley E. Amegah, Edward Nketiah-Amponsah

**Affiliations:** 1 Institute of Statistical, Social and Economic Research (ISSER), College of Humanities, University of Ghana, Legon, Accra, Ghana; 2 Department of Geography, University of North Texas, Denton, Texas, United States of America; 3 Department of Data Science and Economic Policy, University of Cape Coast, School of Economics, Cape Coast, Ghana; 4 Department of Economics, College of Humanities, University of Ghana, Legon, Accra, Ghana; Al Mansour University College-Baghdad-Iraq, IRAQ

## Abstract

**Background:**

In Ghana, breast cancer remains the most common cancer and the leading cause of cancer deaths among women. The cost of treating cancer is huge and poses a great challenge for patients, their families, and health care systems. While comprehensive studies have been conducted on the economic burden of cancers in developed economies such as the EU and the US, there are limited studies in Africa, and Ghana, in particular. This study quantitatively assessed Ghana’s direct and indirect costs of breast cancer treatment.

**Methods:**

Primary data were collected using a questionnaire administered to 217 breast cancer patients at the Korle-Bu and Komfo Anokye Teaching Hospitals, Ghana’s two leading hospitals, and Sweden Ghana Medical Centre. Direct and indirect costs were computed using the Cost-of-Illness Approach. Quantitative analysis was done using multivariate linear regression.

**Results:**

The findings showed that the breast cancer patients studied paid a median amount of Ghana cedis (GHC) 31,021.0 (IQR; 25,262.5–42,147.0), approximating USD 5,500.2 (IQR: 4,477.0–7,469.2 USD) for their treatment within one year of active treatment in 2019. About 61.9% (95% CI: 61.8–62.0%) of this cost was direct cost, while the remaining 38.1% (95% CI: 38.0–38.1%) was indirect cost. Patients who sought care from public facilities for breast cancer paid a median amount of GHC 29,606.3 (USD 5,249.3), while those who sought care from private facilities paid GHC 55,071.2 (USD 9,744.4). Findings from the multivariate linear regression indicate that being married/cohabiting, divorced/separated and having tertiary level education predicted higher cost of breast cancer treatment while patients on retirement and patients in the middle stage (Stage II) of breast cancer diagnoses were associated with lower cost of breast cancer treatment.

**Conclusions:**

The cost of breast cancer treatment poses a significant burden on patients and their families. There is a need for increased public funding for breast cancer treatment to reduce the huge economic burden its treatment poses for patients and their families.

## Introduction

Cancer was expected to rank as the leading cause of death and the most critical barrier to increasing life expectancy in every country of the world in the 21st century [1]. Breast cancer is the most common cancer among women worldwide and is responsible for a significant number of deaths [1]. The treatment of cancer is a complex and costly process and may require a combination of surgical, medical, and radiation therapies. The costs of treating cancer are not limited to the medical expenses incurred during treatment but also include indirect costs such as lost income, enhanced nutrition, transportation, and caregiving expenses. Cancer treatment poses a significant financial burden for both individuals and healthcare systems.

Global spending on anticancer medication was $ 164 billion in 2020 and is projected to increase to $269 billion by 2025 [2]. As far back as 2009, Campbell and Ramsey [3] reported lifetime per-patient costs of breast cancer ranging from $US20,000 to $US 100,000 in the US. Cancer patients in the US incurred out-of-pocket costs of about $3.9 billion and $5.6 billion in 2014 and 2018, respectively [4,5]. Cancer related health care cost in the US was $87.8 billion in 2014, increased to $183 billion in 2015 and is projected to increase to $246 billion by 2030 [4,5]. The total cost of cancer in Europe in 2018 was €199 billion, ranging from €160 per capita in Romania to €578 in Switzerland, of which health care cost constituted €103 billion, informal care costs were €26 billion and productivity loss was €70 billion [6].

In low- and middle-income countries, the cost of cancer treatment is even higher due to a lack of access to affordable healthcare services. For Latin America and the Caribbean, Palacios et al. [7] reported that the weighted average direct medical cost of breast cancer per patient by cancer stage (in 2020 international dollars) was I$13,179 for stage I, I$15,556 for stage II, I$23,444 for stage III, and I$28,910 for stage IV. Ahmad et al. [8 reported an average cost of 29,034.4 Nigerian Naira for cancer patients undergoing chemotherapy in a teaching hospital in Zaria, Nigeria.

Despite the significant impact of cancer on global health, there is limited information available on the direct and indirect costs of cancer treatment in sub-Saharan Africa and Ghana, even though such studies are common in advanced countries [9–11]. However, this information is crucial for policymakers, healthcare providers, and communities to effectively allocate resources and improve access to affordable and effective breast cancer treatment. The purpose of this study is to examine the direct and indirect costs of breast cancer treatment in Ghana, with the aim of providing crucial information for the development of effective policies and interventions to reduce the financial burden of breast cancer treatment. By understanding the costs associated with breast cancer treatment, policymakers, healthcare providers, and communities can work together to improve access to affordable and effective treatment, reduce the financial burden on individuals, families, and healthcare systems, and improve outcomes for women affected by breast cancer.

## Materials and methods

### Study setting

The study adopted a cross-sectional descriptive approach and used a purposive sampling approach to select the Korle-Bu and Komfo Anokye Teaching Hospitals, and Sweden Ghana Medical Centre for the study. These are three major hospitals responsible for the treatment of cancers in Ghana. These hospitals have oncology departments specialised in cancer treatment and serve as referral centres for cancer cases across the country and parts of West Africa. These hospitals are situated in Accra and Kumasi, Ghana’s two largest cities.

The Korle-Bu Teaching Hospital (KBTH) is the leading national referral centre in Ghana and the third largest hospital in Africa. Established in 1923 and with over 1,500 bed capacity, KBTH houses the National Centre of Radiotherapy and Nuclear Medicine, equipped with standard equipment and diagnostic tools for detecting and treating cancers [12]. Komfo Anokye Teaching Hospital (KATH) comes as the second-largest Teaching Hospital in Ghana, after KBTH. Established in 1955 and with a bed capacity of 1,200, KATH serves as a referral centre for 12 out of the 16 administrative regions in Ghana [13]. The Sweden Ghana Medical Centre (SGMC), also located in Accra, with its ultra-modern facilities, serves as the major private centre for cancer care in Ghana. SGMC provides preventive screening, skilful diagnosis, and top-quality treatment options for its patients and is known for its excellent standards, high-quality services, and personalised care [14].

### Selection of study participants

A total of 217 breast cancer patients agreed to participate in the study. Out of this number, 115 sought treatment at the Korle-Bu Teaching Hospital, 71 were at the Komfo Anokye Teaching Hospital, and 31 were from Sweden Ghana Medical Centre. Data on breast cancer prevalence in Ghana are not readily available, which make sample size calculation difficult, if not impossible Therefore, patients were purposively selected using criterion sampling. The criteria for selecting patients included:

- Patients should be above 18 years and be able to give informed consent.
- Patients should have been clinically diagnosed with breast cancer.
- Patients should have commenced biomedical treatment for at least 6 months.
- Patients should not be at the terminal stage of the disease and should be able to hold conversations without any discomfort.

Questionnaires were administered by the researchers to as many people who were available and willing to participate in the study over a period of 6 weeks in each treatment facility. The researchers seized visiting the facilities after saturation was reached and they kept meeting the same respondents.

### Data collection methods and instruments

A questionnaire on direct cost, indirect cost and productivity losses was used. An annotated cost questionnaire for completion by patients put together by Thompson and Wordsworth [15] was adapted for the study. This standardised questionnaire serves as a guideline for researchers doing cost-of-illness studies. Data collection was done through a researcher-administered questionnaire (the questionnaire used has been attached as an S1 Appendix). Data collection took place between the months of March and August 2020. With an introduction from oncology nurses, and after informed consent was sought, questionnaires were administered to patients at the treatment facilities while they waited to be attended to.

### Definition of variables

This study adopted the prevalence-based approach to estimate breast cancer treatment’s direct and indirect costs for a single year (2019) in Ghana. The prevalence approach is useful for estimating the cost of chronic conditions like cancer, which have long duration and follow-up periods. This approach helps measure the past and present cost- of- illness each year [16].

The direct cost was broken down into direct medical and non-medical costs. Cost components addressed under direct medical cost included costs related to physician and oncologist visits, chemotherapy, surgical services, laboratory tests, magnetic resonance imaging, radiotherapy, hospitalisation, and medications used by patients [17]. Direct nonmedical costs included the cost of transportation and special meals for the patients. Indirect cost covered the productivity losses of patients and their caregivers and was estimated using the human capital approach to calculate productivity losses. In-patient cost is the cost incurred due to hospitalisation. Administrative costs cover insignificant costs such as hospital cards and folders, while consultation costs cover costs related to patients’ visits to oncologists [17].

The cost of breast cancer treatment was estimated using the cost of illness framework, which estimates the cost of illness as

C=X+Y, Eq (1)

where C = represents the total cost of illness, X represents the direct cost of illness. Y represents the indirect cost of illness [16]. The direct cost was estimated by summing expenditures linked with seeking treatment, including medical (surgery, chemotherapy, radiotherapy, medications, laboratory investigations) and non-medical expenses (transportation, special meals, and special care). The direct cost was represented as

X=M+N; Eq (2)

M represents direct medical costs, and N represents direct non-medical costs.

The human capital approach [16] was used to estimate the indirect cost of productivity losses to patients and their caregivers in monetary terms.

Productivity loss was calculated by multiplying the total number of productive hours lost (as provided by patients and caregivers) by the daily minimum wage rate of Ghc10.65 in Ghana for 2019 (the year data were collected) [18]. The daily minimum wage rate gives an idea of the minimum income patients lose due to their absence from productive activities especially for those who work in the informal sector.

In calculating the productive hours lost, patients and caregivers provided data on the number of hours they lose every month on treatment, consisting of travel time and waiting time at the hospital, which could have been used for productive activities. Hours lost in a month were multiplied by 12 to ascertain annual productivity losses.

### Indirect cost/productivity loss due to cancer treatment


Y=μ(y1+y2+y3+⋯+yn), Eq (3)

where;

y1 = travel time to obtain health care,

y2 = waiting time at the health facility,

y3 = time spent caring for the sick,

y4 to yn = Any other indirect costs due to cancer

μ = minimum wage rate.

### Data analysis

The data were entered into Microsoft Excel 360 and analysed using STATA 16.1 [19]. The cost of cancer treatment was summarised using descriptive statistics. The main outcome variable, "cost of cancer treatment", data were characterised by right-skewed distribution; hence the natural logarithm of the cost of cancer treatment was used to reduce the impact of the skewed nature of the cost of treatment variable. Multivariate linear regression models were used to identify the factors influencing the cost of cancer treatment in Ghana. The adjusted model included all the variables of interest [20].

### Ethical consideration

Ethical clearance was obtained from the Ethics Committee for Humanities (ECH) of the University of Ghana (ECH011/19-20), the Korle-Bu Teaching Hospital Ethical Review Board (KBTH/MD/G8/20), and the Komfo Anokye Teaching Hospital Review Board (KATH-IRB/AP/023/20). Permission was also sought from research participants after the study’s objectives were explained to them. A written informed consent approved by the ECH was read out and explained to participants, after which they appended their signature. They were also made to understand that their involvement in the research was voluntary. Participants were also assured of confidentiality and anonymity.

## Results

### Background characteristics of study participants

Table 1 presents the demographic characteristics of the study participants. Out of the total of 217 breast cancer patients included in the study, 115 (53%) were seeking treatment at Korle-Bu Teaching Hospital (KBTH), 71 (32.7%) were at Komfo Anokye Teaching Hospital, while 31(14.3%) were seeking care from the Sweden Ghana Medical Centre. The mean age of the study participants was 48.9 ± 11.7 standard deviation, with the majority, 120 (55.3%), of the respondents aged 46 and above. More than half, 131 (60.4%) of the respondents were married or cohabitating, with almost 86 (40.0%) with tertiary education. A vast majority, 205 (94.5%), of the respondents were Christians, and more than half, 125 (57.6%), were from the Akan ethnic group. Most of the respondents, 121 (55.8%), were self-employed, with 103 (47.5%) earning an average monthly income of less than GHC 1,000 in 2019 (i.e., less than approximately USD 177.30/month (at an average exchange rate of 5.64 Ghana cedis to 1 USD in 2019). The average household size of the respondents was five persons, with over half, 124 (57.1%) of the respondents with a household size of 2–5 persons. Most of the respondents, 161 (74.2%) were urban residents, and 153 (70.5%) were diagnosed with middle-stage cancer.

**Table 1:** Background characteristics of study participants.

Variables	Frequency (%)
Mean age (SD)	48.9 (11.7)
Age, In years	
25–35	26 (12.0)
36–45	71 (32.7)
46 and above	120 (55.3)
Marital Status	
Single	32 (14.7)
Married/Cohabiting	131 (60.4)
Divorced/Separated	24 (11.1)
Widowed	30 (13.8)
Highest level of education	
No formal education	18 (8.3)
Primary	47 (21.7)
Secondary	66 (30.4)
Tertiary	86 (39.6)
Religion	
Christianity	205 (94.5)
Islam	12 (5.5)
Ethnicity	
Akan	125 (57.6)
Ewe	41 (18.9)
Ga-Dangme	28 (12.9)
Other ethnics	23 (10.6)
Employment Status	
Employed	180 (82.9)
Retired	21 (9.7)
Unemployed	16 (7.4)
Avg. monthly income (Ghana cedis)	
< 1,000	103 (47.5)
1,000–2,999	99 (45.6)
3,000 and above	15 (6.9)
Household size	
1	13 (6.0)
2–5	124 (57.1)
6 and above	80 (36.9)
Residential Status	
Rural	56 (25.8)
Urban	161 (74.2)
Stage of cancer on diagnosis	
Stage II	64 (29.5)
Stage III (Middle stage)	153 (70.5)
The facility currently receiving treatment at	
Komfo Anokye Teaching Hospital	71 (32.7)
Korle bu Teaching Hospital	115 (53.0)
Sweden Ghana Medical Centre	31 (14.3)

Source: Authors’ data, 2022.

#### Estimate of the overall cost of breast cancer treatment in study

**Fig 1:**
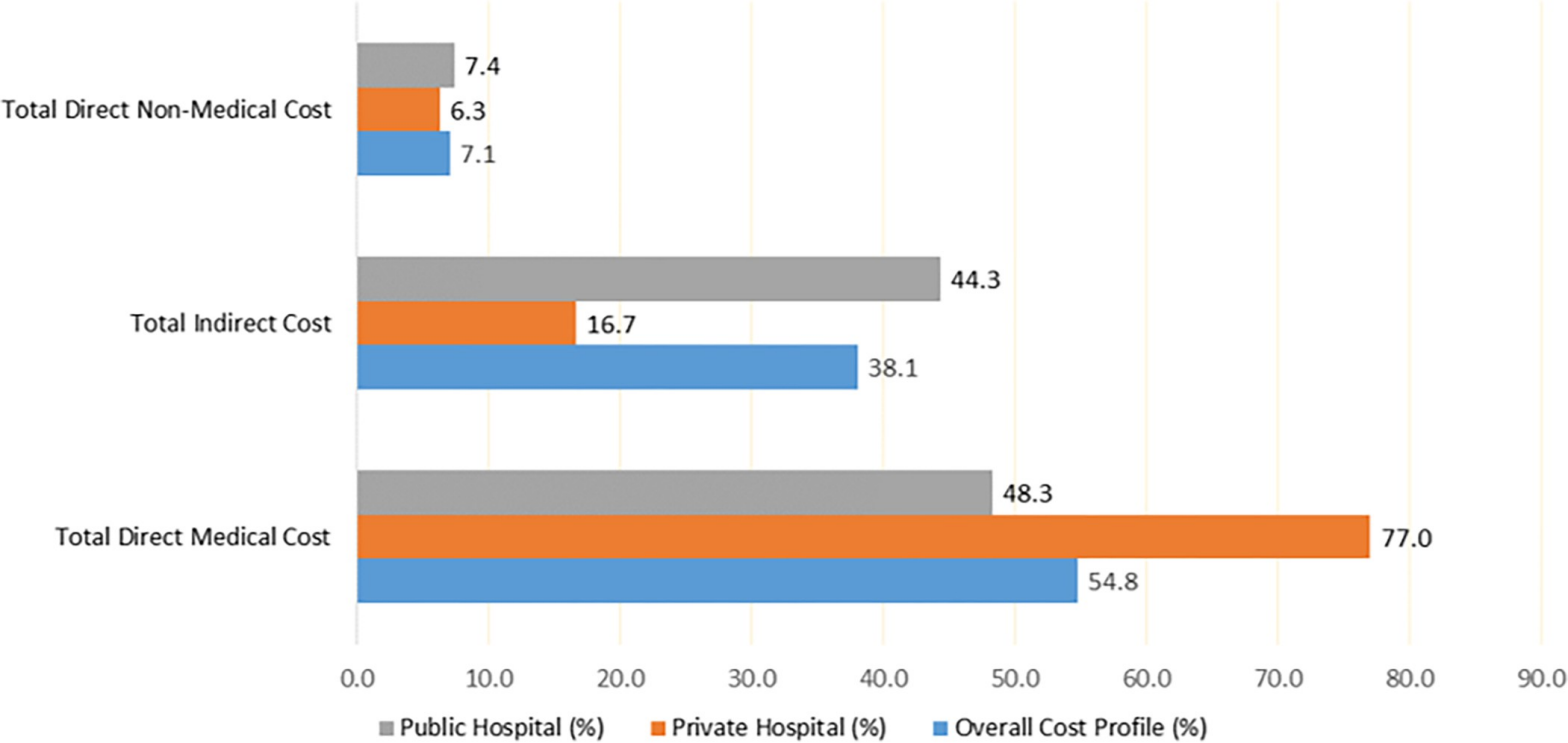
Cost profile of breast cancer treatment.

The total annual cost of breast cancer treatment in the study was estimated at GHC 7,514,935.8 in 2019, approximately USD 1,332,435.4. The median total cancer treatment cost was GHC 31,021.0 (IQR: 25262.5–42147.0 GHC), approximately USD 5,500.2 (IQR: 4,477.0–7,469.2 USD) per patient. The total direct medical cost comprising treatment (surgery, chemotherapy, and radiotherapy), lab tests and imaging, consultations, and medications constituted a major cost component at 54.8% (95% CI: 54.7–54.8). Direct non-medical costs of treatment (transportation, special diet, and care) made up only 7.1% (95% CI: 7.00–7.10) of the total cost. The indirect cost, which includes productivity losses due to illness for patients and their relatives providing care, constituted the remaining 38.1% (95% CI: 38.04–38.11) of the total cost. The cost of cancer treatment varied widely for patients in the study depending on factors such as stage of cancer, type of treatment and patient’s response to treatment. Table 2 presents the overall cost of breast cancer treatment, while Fig 1 shows the composition of direct medical, non-medical, and indirect costs of breast cancer treatment.

**Table 2:** Estimate of the overall cost of breast cancer treatment.

Type of Cost (Ghana Cedis)	Sum (GHC)	Mean	Std. Dev	Median	IQR	Min GHC	Max GHC	Cost %
**Direct Costs**								
**Direct Medical**								
Treatment	2,349,824.0	10,828.7	8,473.3	7,650.0	6682.0, 9900.0	3,800.0	42,800.0	31.3
Investigations Lab tests and imaging	770,371.0	3,550.1	951.2	3,280.0	2880.0, 3980.0	1,940.0	8,000.0	10.3
Consultation	117,170.0	540.0	911.6	190.0	150.0, 230.0	120.0	3,600.0	1.6
Medications	881,374.0	4,061.6	2,250.7	3,360.0	2460.0, 4890.0	1,860.0	15,768.0	11.7
**Total direct medical**	**4,118,739.0**	**18,980.4**	**10,642.90**	**15,070.0**	**13260.0, 19200.0**	**8,815.0**	**57,758.0**	**54.8**
**Direct Non-Medical**								
Transport costs, including Caregiver	231,780.0	1,068.1	1,145.9	600.0	288.0, 1296.0	120.0	6,000.0	3.1
Special diet	258,900.0	3,277.2	1,613.7	3,000.0	2400.0, 4200.0	480.0	7,200.0	3.4
Cost of care	44,160.0	3,680.0	1,440.3	3,720.0	2400.0, 4560.0	1,800.0	6,000.0	0.6
**Total Direct non-medical**	**534,840.0**	**2,464.7**	**2,348.9**	**1,560.0**	**504.0, 3840.0**	**120.0**	**9,720.0**	**7.1**
**Total Direct Cost**	**4,653,579.0**	**21,445.1**	**11,263.1**	**17,590.0**	**15130.0, 21500.0**	**10,090.0**	**63,120.0**	**61.9**
**Indirect Cost**								
Patient Travel Time	94,625.2	436.1	480.8	255.6	191.7, 511.2	63.9	4,089.6	1.3
Absenteeism/Time away from productive ventures	1,751,371.2	9,570.3	8,539.9	6,134.4	4089.6, 10224	1,022.4	46,008.0	23.3
Caretaker travel time	68,554.0	451.0	527.5	255.6	191.7, 511.2	63.9	4,089.6	0.9
Caretaker time spent on care	582,256.8	4,015.6	1,285.5	3,578.4	3067.2, 4473.0	1,278.0	7,668.0	7.7
Patient waiting time	212,339.7	978.5	472.7	766.8	639.0, 1150.2	255.6	2,556.0	2.8
Caretaker waiting time	152,209.8	1,001.4	495.7	798.7	575.1, 1533.6	255.6	2,556.0	2.0
**Total Indirect Cost**	**2,861,356.7**	**13,186.0**	**8,797.5**	**11,459.4**	**7029.0, 16102.8**	**702.9**	**55,337.4**	**38.1**
**Grand Total**	**7,514,935.8**	**34,631.0**	**13,107.4**	**31,021.0**	**25262.5, 42147.0**	**12,251.9**	**89,382.9**	**100.0**

Source: Authors’ data, 2022. *(Inter-Bank Exchange Rate—Month Average, GHC5.6427 = 1 USD, June 2020.

#### Estimate of cost of breast cancer treatment by type of hospital

Tables 3 and 4 show the detailed breakdown of treatment costs in the private and public hospitals. The tables present the sum, mean, median, standard deviation, interquartile range, minimum and maximum and cost profile to further clarify the variation in the cost of treatment. The annual cost of breast cancer treatment in the public hospitals studied was GHC 5,813,116.6 (USD 1,030,694.4), with a median of GHS 29,606.3 (IQR: 24639.5, 35732.4), approximately USD 5,249.3 (IQR: 4368.7, 6335.5) per patient Direct medical cost constituted 55.7% (95% CI: 55.63, 55.72), the direct non-medical cost was 7.4% (95% CI: 7.34, 7.39), and the indirect cost was 44.3%. On the other hand, the total annual cost of breast cancer treatment at the private hospital studied was GHC 1,701,819.1 (USD 301740.9), with a median cost of GHC 55,071.2 (IQR: 55071.2, 49678.0), approximately USD 9764.4 (IQR: 9764.4, 8808.2) per patient, about twice the median cost in the public hospitals studied. The compositions of direct medical, non-medical and indirect costs were 77.0% (95% CI: 76.96, 77.08), 6.3% (95% CI: 6.21, 6.29) and 16.7% (95% CI: 16.67, 16.79), respectively, for patients who sought treatment at the private hospital. Fig 1 presents the cost composition for direct medical, direct non-medical and indirect costs for private and public hospitals.

**Table 3:** Estimate of cost of breast cancer treatment in public hospitals.

Type of Cost Ghana Cedis	Sum (GHC)	Mean	Std. Dev	Median	IQR	Min GHC	Max GHC	Cost %
**Direct Costs**								
**Direct Medical**								
Treatment	1,441,254.0	7,748.7	2,050.5	7,400.0	6600.0, 8700.0	3,800.0	14,600.0	24.8
Investigations Lab tests and imaging	611,606.0	3,288.2	665.1	3,180.5	2800.0, 3705.0	1,940.0	5,985.0	10.5
Consultation	33,320.0	179.1	37.5	180.0	150.0, 210.0	120.0	240.0	0.6
Medications	721,837.0	3,880.8	2,051.4	3,210.0	2310.0, 4590.0	1,860.0	10,470.0	12.4
**Total direct medical**	**2,808,017.0**	**15,096.9**	**3,237.0**	**14,430.0**	**12983.0, 17070.0**	**8,815.0**	**27,375.0**	**48.3**
**Direct Non-Medical**								
Transport costs the patient and accompanying caregiver	159,780.0	859.0	887.1	600.0	240.0, 1200.0	120.0	4,800.0	2.7
Special diet	235,320.0	3,410.4	1,626.0	3,240.0	2400.0, 4200.0	600.0	7,200.0	4.0
Cost of care	33,360.0	3,706.7	1,330.1	3,840.0	2400.0, 4320.0	1,800.0	6,000.0	0.6
**Total non-direct medical**	**428,460.0**	**2,303.5**	**2,316.8**	**1,320.0**	480.0, 3744.0	**120.0**	**9,720.0**	**7.4**
**Total Direct Cost**	**3,236,477.0**	**17,400.4**	**3,930.2**	**16,665.0**	**14782.0, 19280.0**	**10.090.0**	**29,380.0**	**55.7**
**Indirect Cost**								
Patient Travel Time	67,446.4	362.6	299.0	255.6	191.7, 511.2	63.9	2,556.0	1.2
Absenteeism/Time away from productive ventures	1,599,289.2	10,452.9	8,948.1	6,134.4	4600.8, 15336.0	1,022.4	46,008.0	27.5
Caregiver travel time	47,573.5	368.8	334.1	255.6	170.4, 511.2	63.9	2,556.0	0.8
Caregiver time spent on care	539,955.0	4,121.8	1,273.1	4,089.6	3195.0, 4600.8	1,278.0	7,668.0	9.3
Patient waiting time	188,505.0	1,013.5	483.8	862.6	639.0, 1278.0	255.6	2,556.0	3.2
Caregiver waiting time	133,870.5	1,037.8	508.8	894.6	639.0, 1533.6	255.6	2,556.0	2.3
**Total Indirect Cost**	**2,576,639.6**	**13,852.9**	**9,121.0**	**11,608.5**	7412.4, 17125.2	**702.9**	**55,337.4**	**44.3**
**Grand Total**	**5,813,116.6**	**31,253.3**	**9,747.5**	**29,606.3**	**24639.5, 35732.4**	**12,251.9**	**72,300.0**	**100.0**

Source: Authors’ data, 2022. *(Inter-Bank Exchange Rate—Month Average, GHC5.6427 = 1 USD, June 2020.

**Table 4:** Estimate of cost of breast cancer treatment in private hospital.

Type of Cost Ghana Cedis	Sum (GHC)	Mean	Std. Dev	Median	IQR	Min GHC	Max GHC	Cost %
**Direct Costs**								
**Direct Medical**								
Treatment	908,570.0	29,308.7	8,905.2	29,800.0	29800.0, 26600.0	5,570.0	42,800.0	53.4
Investigations Lab tests and imaging	158,765.0	5,121.5	900.1	5,140.0	5140.0, 4930.0	2,910.0	8,000.0	9.3
Consultation	83,580.0	2,704.8	569.8	2,700.0	2700.0, 2250.0	1,800.0	3,600.0	4.9
Medications	159,537.0	5,146.4	3,017.2	4,410.0	4410.0, 3214.0	1,860.0	15,768.0	9.4
**Total direct medical**	**1,310,722.0**	**42,281.4**	**9,818.4**	**43,250.0**	**43250.0, 37200.0**	**16,360.0**	**57,758.0**	**77.0**
**Direct Non-Medical**								
Transport costs the patient and accompanying Caregiver	72,000.0	2,322.6	1,644.6	1,920.0	1920.0, 1200.0	240.0	6,000.0	4.2
Special diet	23,580.0	2,358.0	1,232.2	2,400.0	2400.0, 2160.0	480.0	4,800.0	1.4
Cost of care	10,800.0	3,600.0	2,078.5	2,400.0	2400.0, 2400.0	2,400.0	6,000.0	0.6
**Total non-direct medical**	**106,380.0**	**3,431.6**	**2,345.3**	**3,000.0**	3000.0, 1560.0	**240.0**	**8,400.0**	**6.3**
**Total Direct Cost**	**1,417,102.0**	**45,713.0**	**10,398.7**	**45,940.0**	**45940.0, 40080.0**	**18,916.0**	**63,120.0**	**83.3**
**Indirect Cost**								
Patient Travel Time	27,178.8	876.7	937.6	383.4	383.4, 255.6	63.9	4,089.6	1.6
Absenteeism/Time away from productive ventures	152,082.0	5,069.4	3,599.8	3,961.8	3961.8, 3067.2	2,044.8	20,448.0	8.9
Caregiver travel time	20,980.5	912.2	999.1	383.4	383.4, 255.6	63.9	4,089.6	1.2
Caregiver time spent on care	42,301.8	3,021.6	958.5	2,683.8	2683.8, 2300.4	1,917.0	5,112.0	2.5
Patient waiting Time	23,834.7	768.9	335.1	639.0	639.0, 511.2	383.4	1,789.2	1.4
Caregiver waiting time	18,339.3	797.4	359.3	766.8	766.8, 511.2	383.4	1,789.2	1.1
**Total Indirect Cost**	**284,717.1**	**9,184,4**	**4,992.8**	**7,668.0**	**7668.0, 5878.8**	**2,300.4**	**26,262.9**	**16.7**
Grand Total	**1,701,819.1**	**54,897.4**	**12,486.3**	**55,071.2**	**55071.2, 49678.0**	**25,160.8**	**89,382.9**	**100.0**

Source: Authors’ data, 2022. *(Inter-Bank Exchange Rate—Month Average, GHC5.6427 = 1 USD, June 2020.

#### Factors influencing cost of breast cancer treatment

Results from an unadjusted linear regression model revealed that age, the highest level of education, employment status, average monthly income, and stage of breast cancer on diagnosis significantly predicted the cost of breast cancer treatment. Having tertiary level education (coef. = 0.26, 95% CI: 0.08, 0.44, p<0.001), earning a monthly income of GHC 1,000–2,999 (coef. = 0.10, 95% CI: 0.00, 0.19, p<0.05); and GHC 3,000 and above (coef. = 0.27, 95% CI: 0.08, 0.46, p<0.001) were significantly associated with a high cost of breast cancer treatment. Conversely, patients aged 46 years and above (coef. = -0.15, 95% CI: -0.29, -0.01, p<0.001), being retired (coef. = -0.36, 95% CI: -0.51, -0.20, (p<0.001); unemployed (coef. = -0.19, 95% CI: -0.37, -0.02, p<0.05) and being in the middle stage of breast cancer (coef. = -0.21, 95% CI: -0.31, -0.11, p<0.001) significantly predicted lower cost of cancer treatment.

In the multivariate regression analysis, the model explained about 29.0% (R2 = 0.29) variation in the cost of breast cancer treatment. Being married/cohabiting (coef. = 0.15, 95% CI: 0.01, 0.29, p<0.05); divorced/separated (coef. = 0.18, 95% CI: 0.00, 0.36, p<0.1); widowed (coef. = 0.21, 95% CI: 0.02, 0.41, p<0.05); having tertiary level education (coef. = 0.31, 95% CI: 0.03, 0.43) predicted higher cost of breast cancer treatment among the respondents. Lower cost of breast cancer treatment was significantly associated with retired patients (coef. = -0.35, 95% CI: -0.52, -0.18, p<0.001) and patients in the middle stage (Stage II) of breast cancer on diagnosis (coef = - 0.11, 95% CI: -0.22, -0.00, p<0.05) (Table 5).

**Table 5:** Factors influencing cost of breast cancer treatment (Natural log).

	Unadjusted Model		Adjusted Model
Explanatory Variables	Coefficient	95% CI	Coefficient	95% CI
Constant	** **	** **	10.23***	(9.94, 10.52)
Age, In years				
25–35 (Ref)				
36–45	0.12	(-0.03, 0.27)	0.09	(-0.06, 0.24)
46 and above	-0.15**	(-0.29, -0.01)	-0.10	(-0.25, 0.05)
Marital Status				
Single (Ref)				
Married/Cohabiting	0.07	(-0.07, 0.20)	0.15**	(0.01, 0.29)
Divorced/Separated	0.08	(-0.11, 0.27)	0.18*	(0.00, 0.36)
Widowed	-0.07	(-0.24, 0.11)	0.21**	(0.02, 0.41)
Highest level of education				
No formal education (Ref)				
Primary	0.15	(-0.04, 0.34)	0.10	(-0.09, 0.28)
Secondary	0.11	(-0.07, 0.30)	0.14	(-0.04, 0.33)
Tertiary	0.26***	(0.08, 0.44)	0.23**	(0.03, 0.43)
Religion				
Christianity (Ref)				
Islam	0.08	(-0.13, 0.28)	0.08	(-0.15, 0.31)
Ethnicity				
Akan (Ref)				
Ewe	0.05	(-0.07, 0.18)	-0.01	(-0.13, 0.11)
Ga-Dangme	0.12	(-0.03, 0.26)	0.05	(-0.09, 0.18)
Other ethnics	0.08	(-0.08, 0.23)	0.04	(-0.14, 0.21)
Employment Status				
Employed (Ref)				
Retired	-0.36***	(-0.51, -0.20)	-0.35***	(-0.52, -0.18)
Unemployed	-0.19**	(-0.37, -0.02)	-0.13	(-0.30, 0.05)
Avg. monthly income				
< 1000 (Ref)				
1000–2999	0.10**	(0.00, 0.19)	0.02	(-0.09, 0.13)
3000 and above	0.27***	(0.08, 0.46)	0.11	(-0.09, 0.31)
Household size				
1 (Ref)				
2–5	0.05	(-0.16, 0.25)	-0.06	(-0.25, 0.14)
6 and above	0.09	(-0.12, 0.30)	0.02	(-0.19, 0.23)
Residential Status				
Rural (Ref)				
Urban	0.04	(-0.07, 0.15)	0.01	(-0.10, 0.12)
Stage of cancer on diagnosis				
Stage II (Ref)				
Stage III (Middle stage)	-0.21***	(-0.31, -0.11)	-0.11**	(-0.22, 0.00)
**Model Statistics**				
Mean dependent Var				10.39
R-squared				0.29
F-test				3.96
Akaike crit. (AIC)				134.03
SD dependent Var				0.36
Number of obs				217
Prob > F				0.000
Bayesian crit. (BIC)				

Notes

*** p < .01

** p < .05

* p < .1.

Source: Authors’ data, 2022.

## Discussion

The objective of the study was to estimate the direct and indirect cost of breast cancer treatment for selected patients in selected hospitals in Ghana. The findings point to a very huge cost of breast cancer treatment for patients and their households. Breast cancer patients who were seeking treatment at the public hospitals studied paid a median amount of GHC29,606.3 (USD 5,249.3) per patient while patients seeking treatment at the private hospital studied paid GHC55,071.2 (USD 9,744.4) within a year of active treatment (2019). These results are in conformity with existing literature on the economic burden of cancer treatment globally and in Ghana. For instance, Yabroff et al. [21] reported that cancer care costs 124.5 billion dollars in the United States in 2010 with a projected cost of $157.8 billion dollars by 2020. Luengo-Fernandez and Leal [12] also recorded a cancer cost of €126 billion for the European Union in 2009. Eghdami et al. [17 reported a total cost of US$436,237 and a mean cost of US$3,966 for gastric cancer treatment in Iran in 2016. In Ghana, Hughes et al. [22] reported an average annual cost of breast cancer treatment of GHC6,008.09 per patient in 2006. Gyau [23] reported a low average monthly cost of GHC714.43 for breast cancer treatment. This was because the study was limited to expenditure for a single month, June 2016, which misrepresents the economic burden of breast cancer treatment. Adanu [24] on the other hand estimated an average monthly cost of GHC5,407.4 for breast cancer treatment in 2018.

Regarding the cost composition of breast cancer treatment in this study for both private and public hospitals, 54.8% of the cost consisted of direct medical cost, 7.1% consisted of direct non-medical cost while 38.1% was made up of indirect cost. However, there were differences in the component of cost for patients who sought treatment at public and private facilities. In this study, the direct medical cost of treatment at the private hospital, such as chemotherapy, radiotherapy, laboratory investigations, was very expensive, constituting 77% of the total cost of private care. Indirect cost representing productivity losses was 16.7%, and non-medical cost covering transportation and special diet was 6.3%. For breast cancer patients seeking treatment from a public hospital, direct medical cost constituted 48.3%, non-medical cost made up 7.3%, and indirect cost constituted 44.3%. This finding corroborates existing literature. The medical cost usually contributes to the most significant proportion of cancer treatment. For example, in the European Union, 59% of the total cost of cancer care was made up of health care, while informal care constituted 18%, and productivity losses made up 23% of the total cost [11]. In Eghdami et al.’s [17] work, direct medical costs constituted 59% of the total cost of gastric cancer in Iran while indirect costs constituted 28%, and direct nonmedical costs constituted 13% of the total cost. In Adanu [24], direct medical cost constituted 79.8%, direct non-medical cost was 15.2% and indirect cost constituted only 5% of the cost.

Several factors explain the high component of indirect cost in our study compared to other studies reviewed. First, the majority (86.2%) of breast cancer patients in the study were economically active: 55.8% were self-employed, 30.4% were waged workers and reported the loss of numerous productive hours due to their illness. Hours lost included travelling time, waiting, and seeking medical care at the hospital, and time taken away from productive ventures due to cancer-related weakness. Most of the breast cancer patients studied spent productive hours recovering from surgeries, during hospitalization, and dealing with the side effects of chemotherapy and radiotherapy. All these accounted for the high indirect cost, as seen in this study.

Indirect cost also included hours lost by caregivers who accompanied patients to the hospital and hours spent on providing care for breast cancer patients. Most patients required the assistance of a caregiver at the hospital to run errands like paying bills, purchasing medications, and making inquiries. Such caregivers, especially if they are blood relatives, also provided physical and emotional support to patients at the hospital. For instance, the side effects of chemotherapy rendered most patients weak, hence the need for someone to help them get back to their homes safely. Indirect costs are usually higher for economically active patients due to high-income losses. For instance, Wu et al. [25] found a higher indirect cost among cancer patients who were employed compared to older patients who were not economically active.

Lastly, younger patients (25–45 years), patients with tertiary education and a relatively higher income (GHC1000-3000/USD 177–532 or more), and those with stage II cancer could afford their treatment procedures and comply with recommended special diets. On the contrary older patients (46 years or older), divorced, widowed, unemployed, retired, uneducated, and with low monthly reported significant challenges with paying for treatment procedures and complying with the recommended special diet. They reported gaps in oncology visits and treatment procedures due to financial difficulties. This finding corroborates with Zullig et al. [26] findings that about half (45%) of cancer patients in the US were non-adherent to their cancer medications due to cost. Out of the 45%, 22% took less than the prescribed amount of medication, 25% filled partial prescriptions, and 27% failed to fill their prescription.

It is important to note that this study was a micro-level analysis of the economic burden of breast cancer treatment from the patient’s perspective. Cost estimation consisted of cost of treatment and productivity losses for patients and their caregivers. As such the cost estimation did not include the cost of buildings, equipment acquisition and maintenance, and salaries for health personnel. Further research is needed at the macro level to assess the economic burden of breast cancer treatment on the government and firms in Ghana. There is also the paucity of data on the prevalence rate of breast cancer in Ghana, within the context of fear of stigma, secrecy, and non-disclosure of cancer status by patients. These made it difficult to adopt a random sampling approach in selecting participants for the study. Moreover, there are specific hospitals dedicated to cancer care in Ghana. Therefore, the researchers used a purposive sampling approach to select health facilities and patients for the study. The use of a purposive sampling approach limits the type of analysis that can be run and the extent to which the findings of the study can be generalized. Consequently, our study is not generalizable to the whole of Ghana.

## Conclusions and recommendations

This paper sought to estimate the economic burden of breast cancer for patients in three selected hospitals in Ghana. The cost estimation was comprehensive covering direct and indirect cost. We found that the cost of breast cancer treatment was overwhelming for patients and their families given the relatively low average incomes in Ghana. The financial burden was worse for patients with low socio-economic status such as older patients, widows, divorcees, illiterate, retired and patients with low income. This category of patients had difficulties funding their treatment. The majority of them missed oncology visits, skipped treatment procedures and could not afford their recommended special diet.

It is crucial for government to increase public funding for breast cancer treatment to reduce the economic burden on patients and their households. Targeted financial intervention for older patients, divorced, widowed, unemployed, retired, uneducated, and patients with low income has become critical. Such intervention will reduce financial barriers to accessing treatment and enhance adherence to treatment and cancer survival in Ghana.

It is necessary to decentralise cancer care in Ghana such that diagnosis and treatment occur at the regional level to reduce the cost of transportation and accommodation incurred by patients and their families during treatment. This will also reduce the overload on the oncology units at Korle-Bu and Komfo Anokye Teaching Hospitals.

The most urgent and cost-effective approach to fighting cancer lies in its prevention, which is enhanced through increased awareness, and readily available and highly subsidised screening. There is, therefore, a need for increased educational campaigns to improve cancer awareness and demystify the stigma surrounding cancers in Ghana. The contribution of organisations such as Breast Care International and Pink Africa, and the dedication of the month of October to breast cancer awareness are helpful in this regard.

## Supporting information

S1 Appendix Questionnaire for completion by patients. (DOCX)

## Abbreviations

CI: Confidence interval
ECH: Ethical Committee on Humanities
EU: European Union
GHC: Ghana cedis
GLOBOCAN: Global Cancer Statistics
IRB: Institutional Review Board
IRR: Incident Risk Ratio
KATH: Komfo Anokye Teaching Hospital
KBTH: Korle-Bu Teaching Hospital
LMICs: Low- and Middle-Income Countries
NCDs: Noncommunicable diseases
RC: Reference category
SD: Standard deviation
SE: Standard error
US: United States
USD: United States dollar
