# Supplementary material for: An assessment of the direct and indirect costs of breast cancer treatment in leading cancer hospitals in Ghana
Source: PLoS One. 2024 May 21;19(5):e0301378. doi: 10.1371/journal.pone.0301378 (PMC11108162; doi:10.1371/journal.pone.0301378)
Supplement: S1 Appendix — (DOCX) [file pone.0301378.s001.docx]

**PONE-D-22-31917R1: Questionnaire**
An Assessment of the Direct and Indirect Costs of Breast Cancer Treatment in Leading Cancer Hospitals in Ghana

**QUESTIONNAIRE FOR COMPLETION BY PATIENTS**

**An Assessment of the Economic Burden of Breast Cancer Treatment for Patients and their Households in Ghana****.**

The purpose of this study is to understand the economic burden of breast cancer in Ghana. You are encouraged to complete this questionnaire, as your responses will be useful in achieving the purpose of the study. Your responses will give us an idea of how much you and your family spend on your cancer treatment. This will give us an idea about the cost of living with breast cancer in Ghana. Your answers are important because they will give policymakers and funders an idea of how much the treatment costs you. Your responses will be used for research purposes only and your identity will be hidden during data presentation. The information that you provide will be completely confidential. Your answers will be combined with the answers of other patients involved in the study and reported in such a way that it will not identify you or influence your pattern of treatment. For any information about this study, you can always contact Patience Gyamenah Okyere Asante at the University of Ghana on 0542421707 or [pgokyereasante@gmail.com](about:blank).

**SECTION A**

1. **PATIENT**

*This section is to be completed by the patient with the help of the researcher. Please circle or underline the appropriate answer.*

**1.1 SOCIODEMOGRAPHICS**

*This section covers* *your personal details.*

1. Age ---------------

2. Sex: 1. Male 2. Female

3. Marital status: 1. Married 2. Single 3. Cohabiting 4. Separated 5. Divorced

6. Widowed 7. Other (**please specify**) -----------

4. What is your employment status? 1. Full-time work 2. Part-time work

3. Currently seeking work 4. Homemaker 5. Retired 6. Other (**please specify**)

5. What type of work do you do?................................

6. What is the highest level of education you completed?

1. None 2. Primary 3. secondary 4. College 5. University 6. Post-graduate university 7. Other (**please specify**)

7. What is your ethnicity?

1. Akan 2. Ga/Dangme 3. Ewe 4. Guan 5. Grusi 6. Gurma 7. Mande 8. Other

8. What is your religion?

1. Christianity 2. Islam 3. Traditional 4. Other (**please specify**) …….

9. What is your place of residence? ……..……………………

10. How many people are there in your household (including yourself)?

Number of people -----------

11. What is the monthly income of your household?

1. Ghc 1-499 2. Ghc 500- 999 3. Ghc 1,000-1,499 4. Ghc 1,500- 1999

5. Ghc 2,000 -2499 6. Ghc 2,500-2,999 7. Ghc 3,000-3.499 8. Ghc 3,500-3999

9. Ghc 4,000-4,499 10. Ghc 4,500- 4999 11. Other (please specify) ……….

12. What job does your spouse do?...............................................

**1.2 CANCER DIAGNOSIS**

*This section asks questions about your cancer diagnosis.*

12. When were you diagnosed with cancer?

1.Year and month………………………………. 2. I don’t remember

13. What type of cancer were you diagnosed with?

1. Breast cancer 2. Cervical cancer

14. What was the stage of the cancer at diagnosis

1. Early stage (I, II) 2. Middle stage (III) 3. Advanced stage (IV, V)

15. Where (name of hospital) did you start your cancer treatment?

1. Sweden Ghana Medical Centre 2. Korle-Bu Teaching Hospital

3. Komfo Anokye Teaching Hospital 4. Peace and Love Hospital.

5. Other **(Please specify) …………………………**

16. Where (name of hospital) are you currently receiving cancer treatment?

1. Sweden Ghana Medical Centre 2. Korle-Bu Teaching Hospital

3. Komfo Anokye Teaching Hospital 4. Peace and Love Hospital.

5. Other **(Please specify) ……………………………………**

**1.3 DIRECT MEDICAL COSTS**

*This session covers the direct cost involved in your treatment including in-patient and out-patient cost, cost of medications, medical consultations, and other treatment costs.*

**1.3.1 Treatment cost**

17. *Please select treatment options you have used and indicate the number of treatment sessions and the cost involved.*

| **Type of treatment** | **Number of treatment sessions** | **Cost per treatment session** | **Total cost of treatment** | **Paid by** |
| --- | --- | --- | --- | --- |
| Surgery |  |  |  |  |
| Radiotherapy |  |  |  |  |
| Chemotherapy |  |  |  |  |
| Brachytherapy |  |  |  |  |
| Hormone Therapy |  |  |  |  |
| Targeted Therapy |  |  |  |  |
| **Grand Total** |  |  |  |  |

**1.3.2 Medication costs**

18*. Please indicate your prescribed medication and their cost*

| **Name of medication** | **Cost (Ghc)** | **How often do you take this medication in a week/month** |
| --- | --- | --- |
|  |  |  |
|  |  |  |
|  |  |  |
|  |  |  |

**1.3.3 Other direct costs**

19*. Please indicate any other direct cost due to your illness.*

| **Item** | **Number of sessions** | **Cost per session** | **Total cost** | **Paid by** |
| --- | --- | --- | --- | --- |
| Consultation cost |  |  |  |  |
| Laboratory tests:  Biopsy  Full blood count  X-ray  CT Scan  Mammogram  MRI  Kidney function test  Liver function test  Potassium test  Others **(please specify)** |  |  |  |  |
| Alternative treatment (Herbal, Chinese etc.) |  |  |  |  |
| Other costs **(Please specify)** |  |  |  |  |
| **Grand Total** |  |  |  |  |

**1.4 DIRECT NON-MEDICAL COST**

**1.4.1 Travel and Time Costs**

*This part of the questionnaire asks about the costs of your travelling to the hospital/health facility*

20. How do you travel to and from the hospital?

1. Public transport 2. Taxi 3. Private car 4. Motorbike 5. Combination (**please specify**) ……………………………. 6. Other (**please specify**) …………………

21. How much do you spend for one round trip to the hospital?

Ghc…………

22. How long does it take to travel to and from your home to the hospital?

Number of hours ………………. Number of minutes……………...

23. How many times in a month do you go to the hospital?

Number of days in a month………….

24. How many hours or minutes do you spend at the hospital when you come for treatment? This includes waiting time and time with your doctor or time at the lab.

Number of hours…………………… Number of minutes……………

25. How many days or weeks does your treatment take?

| **Treatment session** | **Number of weeks or days** |
| --- | --- |
| Surgery |  |
| Radiotherapy |  |
| Chemotherapy |  |
| Brachytherapy |  |
| Hormone therapy |  |
| Other **(Please specify)** |  |

26. Does anyone follow you to the hospital for your treatment?

1. Yes 2. No 3. Sometimes

**1.4.2 Special meals and care costs**

27. Are you on a special diet because of your illness?

1. yes 2. No **If no, skip question 27 and 28.**

28. If yes, is the special diet more expensive than your regular meals before cancer diagnosis?

1. Yes 2. No 3. Don’t know **If no or don’t know, please skip question 28.**

29. If yes, how much more do you spend on a special diet?

Ghc………………………….

30. Do you need someone to provide care for you, your children or to do house chores because of your illness?

1. Yes 2. No, I still do these things myself 3. Sometimes

**If no, skip questions 30 and 31.**

31. Do you pay for such services?

1. Yes 2. No 3. A relative does care work for me 5. Other **(Please specify)**

**If no, please skip question 31**

32. If you pay for care work how much do you pay?

Ghc…………………….

**1.5 PRODUCTIVITY LOSSES DUE TO ILLNESS**

*The next section of the questionnaire asks about how your cancer condition or treatment has affected both your paid work (if you are currently in employment or business activity if self-employed) and your unpaid work. By unpaid work we mean activities such as household chores, shopping, caring for children and voluntary work, non-leisure activities that involve your time but for which you do not get paid.*

33. What work were you doing before your cancer diagnosis?

1. waged worker 2. Self-employed 3. Home keeper 4. Unemployed 5. Other

**If self-employed or home maker, skip to question 38.**

34. If you are a waged worker, what sector do you belong to?

1. Public sector 2. Private sector 3. Other **(please specify)** ……………

35. If you are a waged worker, is your employer aware of your cancer diagnosis?

1. Yes 2. No 3. They know I am sick but don’t know it is cancer

36. Has your illness affected your productivity at work?

1. Yes 2. No 3. To some extent

37. How do you make time for your treatment?

1. I have excuse duty 2.I have taken leave with pay 3. I have taken leave without pay

4. I have stopped working altogether 5. Other **(please specify)** ……………

38. How many days do you absent yourself from work in a month because of your work?

Number of days…………………………...

**If waged worker, skip questions 38-47.**

***Self-employed or home worker***

39. If you are self-employed or a home worker, how many hours did you work in a week before your diagnosis?

Number of hours……………………

40. How much did you earn monthly at that time?

Ghc……………………………

41. Are you doing the same job now?

1. Yes 2. No 3. Other (Please specify) …………………

**If no, please skip to question 47.**

42. If you still work, has your output reduced because of your illness?

1. Yes 2. No 3. Other **(Please specify)**

**If no skip question 42**

43. If yes, how many working hours do you lose weekly because of your illness?

Number of hours…………….

44. Has your income reduced because of your illness?

1. Yes 2. No 3. Other **(Please specify)**

**If no, skip question 44.**

45. If your income has reduced, how much do you lose every week because of your illness?

Ghc………………………….

***Those who have stopped working***

46. If you have stopped working, how many hours do you lose in a week due to your illness?

Number of hours…………….

47. If you have stopped working, how much do you lose every month because of your illness?

Ghc………………………………

48. Why did you stop working?

1. I get very weak, so I am unable to work 2. I want to focus on my treatment now

3. I was laid off 4. Other **(please specify)** …………………

**Caregivers loss of productivity**

49. If a relative provides you with care without payment, how many hours do they spend in providing care for you in a week?

Number of hours……………………………………

50. What work does your caregiver do?

1. waged worker 2. Self-employed 3. Home keeper 4. Unemployed 5. Student

6. Other …………………

**If home keeper or unemployed, please skip questions 48 and 49.**

51. Does your caregiver take time off to provide care services?

1.Yes 2. No 3. Sometimes

52. If yes, how much do they lose in a week or month?

Ghc………………….

Thank you for taking the time to respond to these questions.
